# Release of VOCs, Gasses, and Bacteria from Contaminated Landings and Creeks of Ogeechee River Basin

**DOI:** 10.3390/ijerph191610210

**Published:** 2022-08-17

**Authors:** Victoria Clower, Melanie Sparrow, Atin Adhikari

**Affiliations:** 1 Department of Biostatistics, Epidemiology, and Environmental Health Sciences, Jiann-Ping Hsu College of Public Health, Georgia Southern University, Statesboro, GA 30460, USA; 2 Ogeechee Riverkeeper, P.O. Box 16206, Savannah, GA 31416, USA

**Keywords:** air quality, water quality, trash waste, gasses, bacteria, environmental health, *Escherichia coli*, anthropogenic pollution

## Abstract

River landings are common public grounds, visited by many people every day. The aftermath of visiting these places may be unsettling since much trash is left behind and scattered throughout. The litter collects and with each rain or high wind, it has a better chance of ending up in our streams, rivers, creeks, and eventually our oceans. The main purpose of this study was to measure both air and water quality throughout the Ogeechee River basin in South Georgia to determine how each was impacted by trash. Ammonia, methane, and volatile organic compounds (VOCs) along with temperature and humidity were also measured for air quality. Water quality parameters for this study were derived from the Georgia Adopt-A-Stream method. Conductivity, dissolved oxygen, pH, water temperature, and *Escherichia coli* counts were taken every two weeks at three locations: Rocky Ford Landing along the Ogeechee River, Rocks River Landing on the Canoochee River, and Little Lotts Creek located in the center of Statesboro, Georgia. Each Monday, from 17 January 2022 to 17 May 2022, selected air pollutants were sampled ten times at each location. The data show higher trends in air and water pollution where trash is found—the highest with Rocks River Landing and Little Lotts Creek showing trends supporting the hypothesis that trash may impact air and water quality in these areas. The potential contamination of river landings and creeks may contribute to increased levels of airborne and waterborne gas levels and microbial loads near the river water surfaces.

## 1. Introduction

River landings are common public grounds, visited by many people every day. River landings or boat landings are openings along the banks of rivers where individuals can enter the river with ease. Landings can sometimes have concrete slabs to enable easier access for fishermen and their boats or may not be paved at all. Throughout the United States, these sites are most commonly visited for boating, picnics, and other activities such as parties that may contain alcohol or illegal activities, since many are remote from the inner cities or in locations where public roads are not in direct sight. The aftermath of visiting these places may be unsettling since much trash is left behind and scattered throughout. The litter collects and with each rain or high wind, it has a better chance of ending up in our streams, rivers, creeks, and eventually our oceans. The quality of the water would generally be the first concern; however, the objective of this study is to measure both air and water quality parameters to determine how pollution is impacted by trash that contaminates these sites. This is not just a local problem and remains a prominent issue related to public health concerns throughout many watersheds and rivers of the U.S. [1].

Historical trends have shown that the prevalence of trash has increased exponentially since the early 2000s, with take-out food and single use ware making the top of the list [1] (p. 12). Across the U.S., cities have tried to manage and collect litter and garbage; however, most trash still finds its way into stormwater drains and into nearby rivers. In the state of Maryland, for example, food packaging waste and plastic bottles were listed as the second and third highest categories; meanwhile, food service ware leads as the largest category. Throughout many cities of California—including San Francisco, San Jose, and Santa Cruz—stormwater drains and rivers were constantly filled with food service waste with consistent findings of beverage containers, plastic utensils, and other scattered debris [1]. 

Each year, millions of tons of trash are dumped into our waterways, both soluble and insoluble. In fact, according to the organization called Keep Statesboro-Bulloch Beautiful (KSBB), 267.8 million tons of solid waste are generated by the public alone [2]. Trash can degrade the soil, leach toxic chemicals, and be broken down, deformed, and bioaccumulate in fish, birds, and other animals [3]. Ogeechee Riverkeeper—a nonprofit organization in Georgia, USA—is known for their involvement in improving, protecting, and preserving the water quality of the Ogeechee River basin and offering education on Georgia’s watersheds, monitoring programs, and volunteering events to the public [4]. This organization and its partners look to reduce the impact that waste has on the environment through donating and recycling when possible. KSBB is among the many partners of the Ogeechee Riverkeeper and aids in recycling using vendors such as “Green Rock Recycling, formerly known as Boro Recycling” [2]. 

Some environmental pollutants released through the degradation of trash and waste are gasses such as ammonia and volatile organic compounds (VOCs), which can be released into the air during the decaying process after being exposed to sunlight [5]. Methane gas can also be released during the microbial biodegradation of plastics [6]. Many plastics contain a chemical known as polyethylene terephthalate, which is used mostly in cosmetics, soft drink containers, and water bottles. This plastic is usually clear, smooth, and solvent-resistant and begins to soften at 80 °C. When exposed to higher temperatures, this type of plastic can release acetaldehyde, antimony, and phthalates, which are very harmful [7]. Other issues with plastics in the waterways are microplastics and perfluoroalkylated substances (PFAS). PFAS are contaminants that may have carcinogenic, endocrine-disrupting, and reproductive effects and have already been found in human blood and breast milk [8]. Macroplastics do not readily biodegrade in the environment but rather erode via chemical and physical processes, and when plastic trash, textiles, and other pollutants are thrown into the water system, they are exposed to ultraviolet light which can break down to microplastics which are smaller than 5 mm [8] (p. 1). When trash is dispersed by wind or stormwater, macro, micro, or nanoparticles can form through abrasion, the act of ripping and tearing forced by high pressure from other objects; high winds; or photooxidation [1]. Out of all types of plastic, research has shown that polyethylene has the highest concentration among other types of plastic in the environment. Since many plastic types can help accumulate PFAS in the environment, it is essential to eliminate plastic trash in waterways. This form of plastic dominates among other items due to its durability to last for long periods of time in the environment. 

The surface water chemical parameter, conductivity, has a World Health Organization (WHO) maximal permissible level of 900 siemens per meter (S/m), which determines the flow of ions in electrolytes of flowing water [9]. Dissolved oxygen measures the oxygen level in the water and how rich or reduced the oxygen is. Temperature and relative humidity are great associations between gas release, and bacteria counts for both water and air quality. These parameters also play a role in the decaying process of trash, especially plastics, and may pose a risk to humans visiting these sites or using these landings for recreational purposes by controlling the rate at which reactions occur [10]. When temperatures are high, oxygen levels decrease, and when temperatures are low, dissolved oxygen in water tends to be higher. Levels of pH help determine how acidic or basic a water body is connected to other reactions that may or may not occur as well including photosynthesis. The secondary bacteria criteria for fishing and recreational use should never exceed 126 colony counts per 100 mL at one site over a month’s time [11].

Air and water quality parameters are connected, and trash is inevitable with populations continually rising along the southeast coast of USA. Air and water monitoring at these locations are crucial to determine the impact that human activities have on not just human safety but also plants and animals living in these environments. This study showcases the ever-growing concern that trash decomposition can have adverse impacts on the waterways and its surrounding environments and also hopes to propose solutions by implementing the use of trash booms at more monitoring sites. It also provides essential information about the possible damage that can be caused by anthropogenic, or human-inflicted, pollution. By following multiple sites throughout the Ogeechee River basin, we can see that results may differ by popularity, concentration of trash on the land versus along the water’s surface, and from events taking place downstream of these sites.

## 2. Materials and Methods

Bacteria and gas samples were taken at three locations of Ogeechee River basin: Rocky Ford Landing between the time frame of (8 a.m.–10 a.m.), Rocks River Landing between the times of (11 a.m.–1 p.m.), and Little Lotts Creek between the times of 2 p.m. and 4 p.m. from 17 January 2022 to 17 May 2022. An SKC Biostage Aerosol Sampler (Single-Stage Viable Cascade Impactor) and SKC Quick Take Pump along with the inlet adapter with tubing and a biostage mounting bracket were used to collect six air samples for airborne bacterial and a field blank at each location. Six bacteria samples were collected every two minutes on a Tryptic Soy Agar (TSA) medium plate. This instrument is used by taking apart the biostage sampler which consists of three parts: the inlet, the jet classification stage, and the sampler base which is where the agar plates would rest. Once the impactor was reassembled with a TSA plate, the SKC Quick Take Pump was set to 2 min and pulled in the air at approximately 28.3 L/min. The Biostage Impactor was wiped cleaned with 70% ethyl alcohol between each sample to ensure minimum contamination, and all TSA plate lids were placed in prelabeled ziplock bags during sampling and immediately placed back on the TSA plates post sampling.

Other equipment used in the air sampling process was the ppbRAE 3000 (VOC monitor), Aeroqual Series 200/300/500 (methane monitor), and the GasAlert Honeywell Extreme Single Gas Detector (ammonia monitor), which continually took in air and was checked every two minutes. Overall, a total of twenty minutes of sampling took place at each site with air samples being collected every two minutes with a total of ten gas samples per location. Ammonia and methane were read in parts-per-million (ppm); meanwhile, VOCs were calculated in parts-per-billion (ppb). Meanwhile, the TSA bacteria air samples were collected using an active pump, plates were incubated for colony growth, and finally the colony forming units (CFU) per cubic meter (m^3^) of air or CFU/m^3^ values were calculated after positive hole conversions. One field blank per site was taken for bacterial air sampling. Field blanks were placed and enclosed in a pre-sanitized impactor; however, no pump was used to bring in outside air. This helped ensure that the equipment quality was in good condition and limited sampling error. 

Water quality parameters for this study were derived from the Georgia Adopt-A-Stream method, which is based on a citizen science project where volunteers can choose to adopt a river landing, creek, stream, or estuary in their community and monitor that site [12]. Free training sessions are also offered to test for chemical, bacterial, or macroinvertebrates where a certificate can be obtained and last for a single year until recertification is required. Upon sampling, all water parameters are then inserted into their system through a data submission form where researchers analyzed the data and checked for accuracy. The water quality parameters included in the chemical kit were conductivity, dissolved oxygen, pH, water temperature, and *E. coli* counts in the units of CFU/100 mL. If any warning signals appear throughout the process, the Adopt-A-Stream members may request more information. If point source pollution was ever discovered in a community, the Environmental Protection Division (EPD) will be connected and should resolve the situation. 

**Scheme 1:**
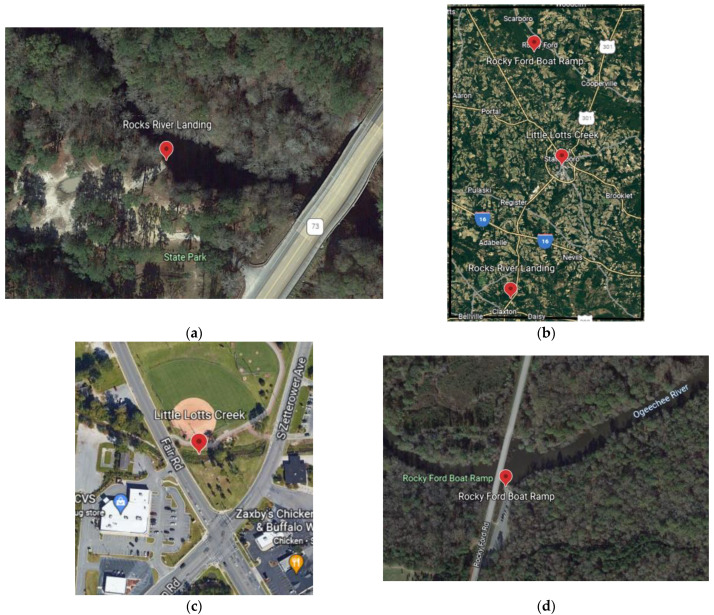
Aerial view of Rocky Ford Boat Ramp along Ogeechee River in Statesboro, GA (**a**). Aerial view of all sampling sites to showcase distance from one location to the other (**b**). Aerial view of Little Lotts Creek in Statesboro, GA (**c**). And Rocks River Landing in Evans County GA (**d**).

All water parameters were taken every two weeks at Little Lotts Creek, Rocky Ford Landing, and Rocks River Landing. River landings were chosen based on their location in the Ogeechee River Watershed. An aerial view of all locations together can be seen in Scheme 1a; Rocky Ford Landing is located north of Statesboro Georgia as seen in Scheme 1b. Meanwhile, Rocks River Landing is located along the Canoochee River in Claxton, Georgia, with an aerial view seen in Scheme 1c. Finally, the Little Lotts Creek site is located in the center of Statesboro and can be seen in Scheme 1d. The Canoochee River is Ogeechee River’s largest tributary, and Little Lotts Creek leads to the Canoochee River and then into Ogeechee River, so they are all connected before they reach the Atlantic Ocean. 

To sample *E. coli*., waders or boots were worn to enter the river at least knee level where free flowing water was moving. Gloves were worn to open one whirl-Pak sampling bag with pre-written identification, ID, name, date, and site number. Water from upstream of the river was collected away from the body to ensure no contamination and filled to 2/3 full. All samples were kept on ice during transportation, and water and bacterial air samples were kept in separate coolers. Once in the lab, 1 mL of water was taken from each Whirl-Pak sampling bag using a pipet and placed in the center of bacteria plates. This procedure was repeated two additional times for a total of three plated water samples per Whirl-Pak bag, ensuring to invert the water between each sample. One lab blank was also completed using distilled water to represent each site. Plates were placed in the incubator between 35 ± 1 °C and left for 24 h ± 1 h. 

Trash was collected for a minimum of 1 h and max of 2 h every 2 weeks at each location depending on its need. Trash analysis was done immediately prior to calculating exact litter numbers. This was completed by emptying all trash bags of their contents and counting each item picked up within that time frame. If more than one bag was picked up and full of trash, random selection took place to count items to represent a site. At Little Lotts Creek, trash booms were located. The trash booms were referenced from the Osprey Initiative whose mission is to collect trash and litter using new technology [13]. Trash booms are floating devices that remain on top of the water and are connected together on a long rope. Using this technique helps to stop the trash from continuing downstream of the creek and enables volunteers to collect it. Two trash booms were located at this location, within 20–30 feet from each other. The second boom was installed to catch any trash that may have escaped the first one and is located at the intersection of Zetterower Ave and Fair Rd. Little Lotts Creek was also a previously established location for the Ogeechee Riverkeeper. The program known as “Don’t Litter Lotts” is partnered with both the KSBB and the City of Statesboro to collect Adopt-A-Stream chemical and bacterial samples as well as trash on a bi-weekly to monthly basis. Two members/volunteers were already monitoring this site upon the start of the study. 

## 3. Results

### 3.1. Air Quality Impact

**Figure 1:**
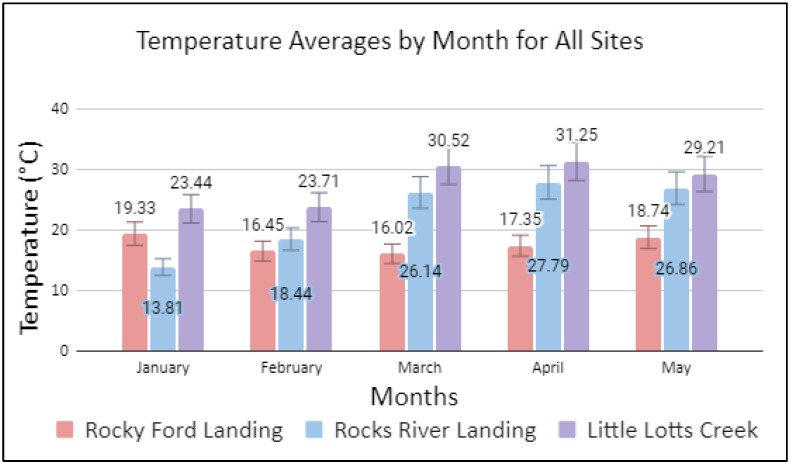
Air Quality—Temperature (°C) Monthly Averages.

**Figure 2:**
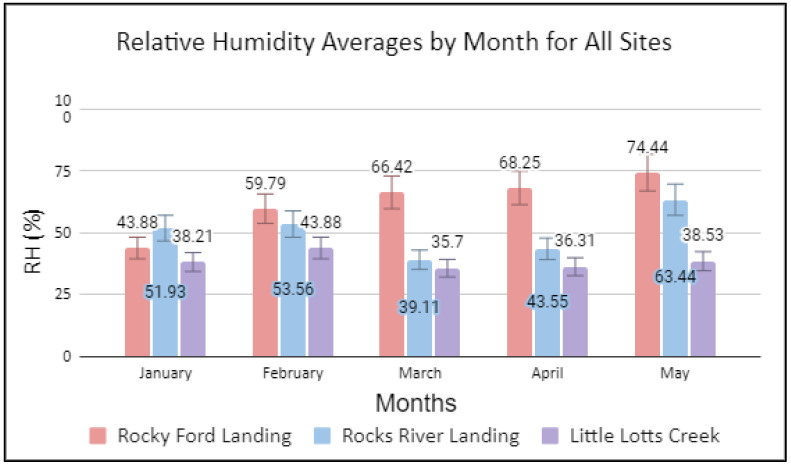
Air Quality—Humidity (%) Monthly Average.

After five months and 15 weeks, a total of 90 bacterial samples were collected and 150 gas samples were analyzed at each site. For water quality, a total of 7 weeks were followed at Rocky Ford and Rocks River Landings; however, at Little Lotts Creek, only two full weeks of all parameters were collected since the start of the study. Trash was collected every two weeks from each site and at least once a month. At Rocky Ford, the total amount of trash collected throughout the study was 19.64 kg; meanwhile, Rocks River Landing and Little Lotts Creek had a total trash collection of 69.3 kg and 48.85 kg, respectively. Since the Rocks River and Little Lotts Creek had the highest counts of trash, in theory, the air and water quality would also be most impacted if our hypothesis was correct. Figure 1 and Figure 2 illustrate the average temperatures in degree Celsius and relative humidities for each month that was monitored at each site. On average, Rocky Ford had the lowest temperature with an average temperature and relative humidity (RH) of 17.12 °C and 61% RH, respectively, due to having the earliest sampling time. This was followed by Rocks River landing and Little Lotts Creek, which had higher temperatures and a lower percentage of humidity that averaged at 21.83 °C and 48.0% RH at Rocks River Landing and 27.33 °C and 38.5% RH at Little Lotts Creek throughout the entirety of the study.

**Figure 3:**
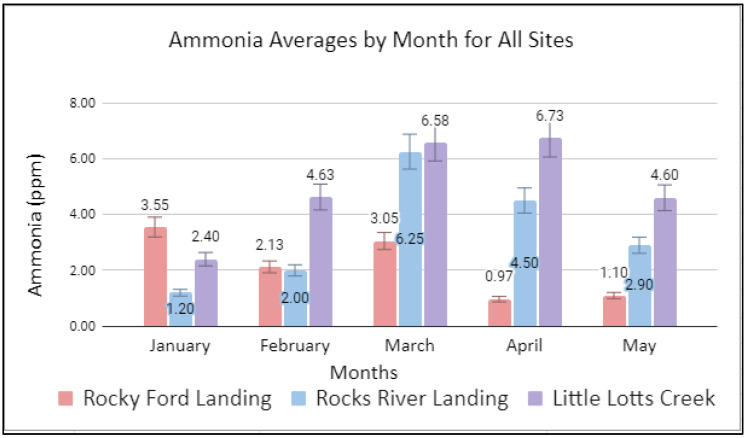
Air Quality—Ammonia (NH_3_) Gas Sample Measurements.

**Figure 4:**
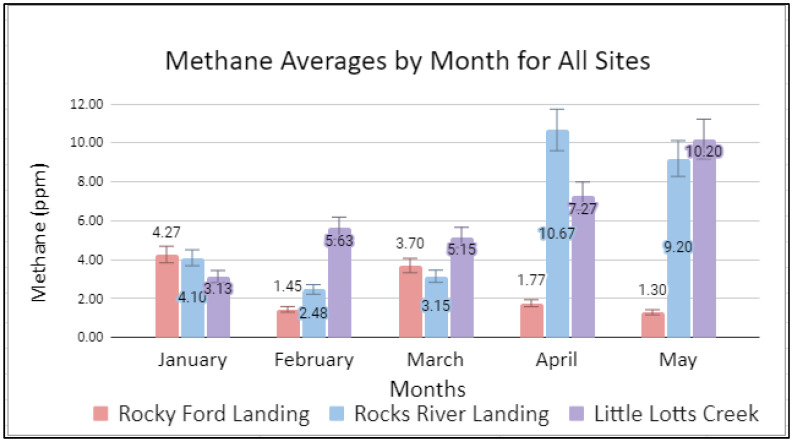
Air Quality—Methane (CH_4_) Gas Sample Measurements.

**Figure 5:**
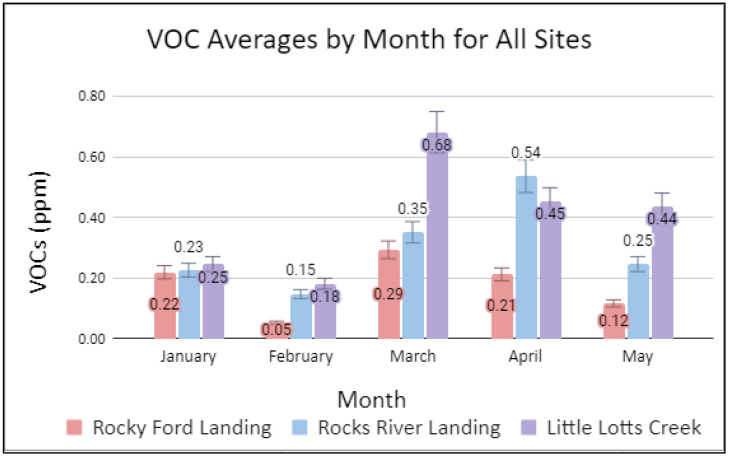
Air Quality—Volatile Organic Compounds (VOCs) Measurements.

**Figure 6:**
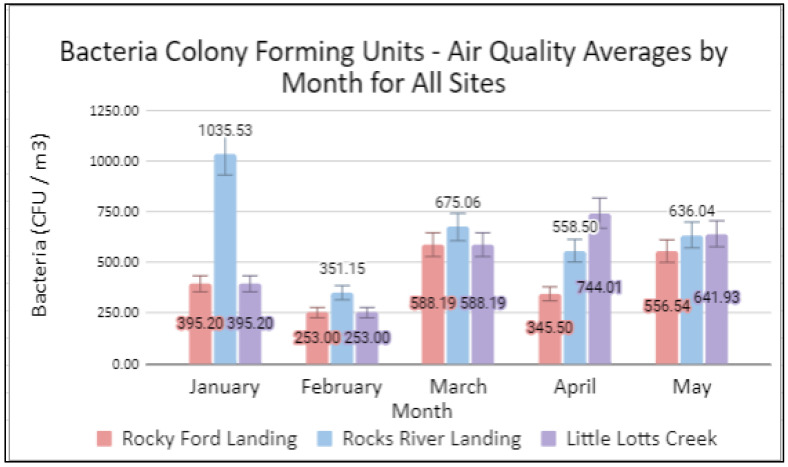
Air Quality—Total Culturable Bacterial Concentrations.

Ammonia, methane, VOCs, and bacterial samples were also collected. Figure 3, Figure 4, Figure 5 and Figure 6 demonstrate a trend showcasing Rocky Ford once again having the lowest total averages for all gas samples. For Ammonia, methane, and VOCs, Rocky Ford total averages followed by their standard deviations were 2.3 ± 2.3 ppm, 2.7 ± 3.3 ppm, and 0.19 ± 0.13 ppm, respectively. Rocky Ford’s bacterial samples also had the lowest colony-forming units per cubic meter averaging at 407.7 ± 427.3 CFU/m^3^. These can be seen compared to the other two locations in Table 1. At Rocks River Landing, ammonia, methane, VOCs, and bacteria had the second highest gas readings with total averages of 3.7 ± 3.3 ppm, 5.1 ± 4.7 ppm, 0.31 ± 0.18 ppm, and 634.9 ± 851.9 CFU/m^3^. Little Lotts Creek had the highest averages for all gasses and colony-forming units per cubic meter. For ammonia, methane, and VOCs, its total averages were 5.3 ± 3.8 ppm, 5.6 ± 3.5 ppm, and 0.38 ± 0.24 ppm, respectively. Little Lotts Creek’s bacteria count averaged at 666.3 ± 1034.1 CFU/m^3^. 

### 3.2. Water Quality Impact

**Figure 7:**
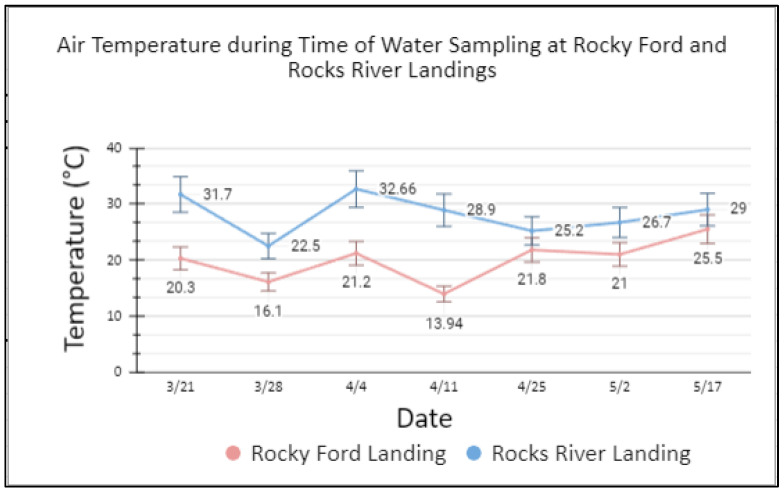
Water Quality—Air Temperature in Degree Celsius from March–May 2022.

**Figure 8:**
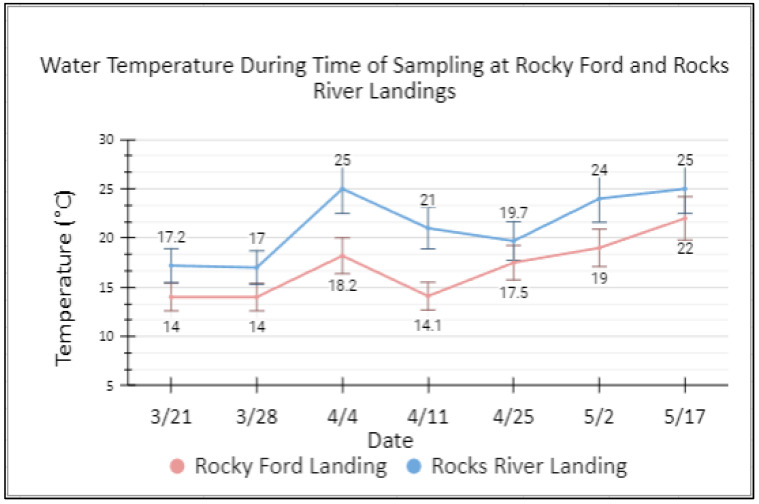
Water Quality—Water Temperature in Degree Celsius from March–May 2022.

**Figure 9:**
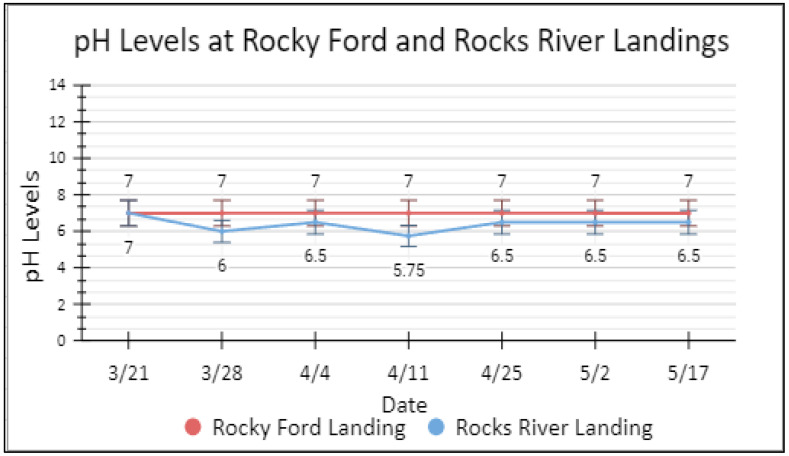
Water Quality—pH Level Measurements from March–May 2022.

**Figure 10:**
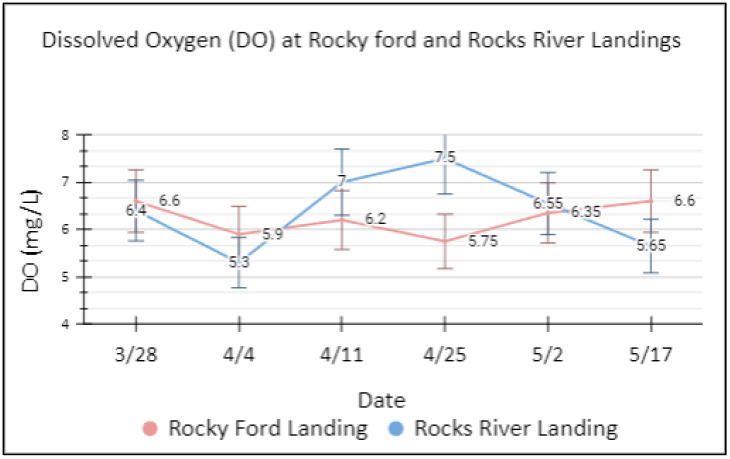
Water Quality—Dissolved Oxygen Level Measurements from March–May 2022.

**Figure 11:**
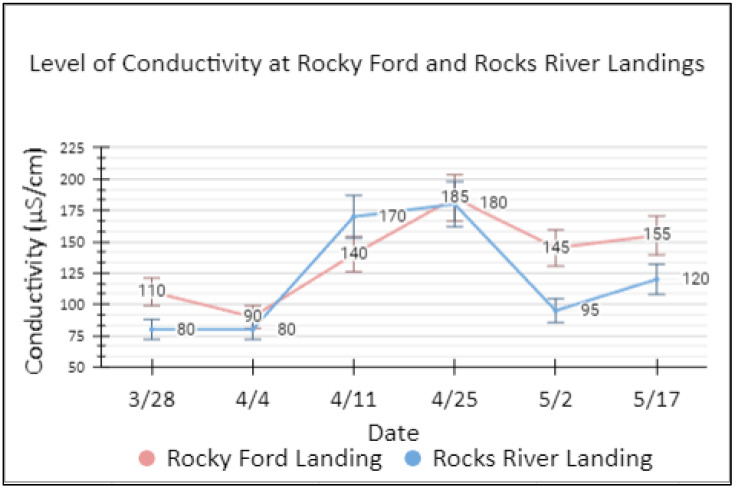
Water Quality—Level of Conductivity from March–May 2022.

**Figure 12:**
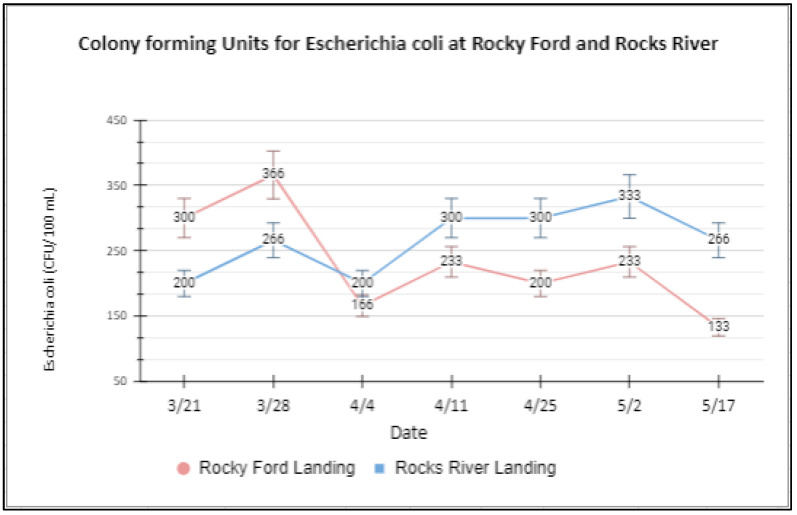
Water Quality—*Escherichia coli* Measurements from March–May 2022.

Water samples were not able to be collected for the months of January and February at the two river landings until an Adopt-A-Stream certification was completed and all chemicals were updated, which left only three months of sampling available for this study. Additionally, Little Lotts Creek was not sampled repeatedly on the same day as other samples were taken. In terms of water quality parameters, water temperature and air temperature at time of sampling were taken. Conductivity, pH, DO, and bacterial testing for *Escherichia coli* were also sampled. Figure 7 and Figure 8 indicate the air and water temperature weekly averages at all sites from 21 March to 17 May 2022. A total of seven samples were collected for both Rocky Ford and Rocks River and are shown in these figures. For Rocky Ford, both the air and water temperatures were lower than at Rocks River with an overall average of 20.69 °C and 18.16 °C, compared to Rocks River Landing whose total air and water quality averages were 28.08 °C and 21.3 °C, respectively. This once again is due to the difference in sampling times which were 8–10 a.m. at Rocky Ford and 11 a.m.–1 p.m. at Rocks River Landing. Figure 9, Figure 10, Figure 11 and Figure 12 show the monthly averages for pH, conductivity, dissolved oxygen, and *E. coli*. 

At Rocky Ford Landing and Rocks River Landing, the pH, DO, and conductivity levels averaged at 7, 6.16 ± 0.35 mg/L, and 143 ± 33.6 µS/cm and for Rocks River at 6.5 ± 0.4, 6.4 ± 0.82 mg/L, and 80 ± 44.54 µS/cm, respectively. Rocky Ford had the lowest *E. coli* counts averaging at 193.2 ± 79.42 CFU/100 mL, while Rocks River Landing had a total *E. coli* count average of 233 ± 50.9 CFU/100 mL. At Little Lotts Creek, water samples were not taken regularly nor was each parameter sampled each time. Table 2 indicates the dates and water parameters that were sampled; meanwhile, Table 3 lists all total averages and standard deviations for all locations and water parameters. Even though Little Lotts did not have all parameters recorded on a regular basis, it still showed the highest *E. coli* counts, which were 316.3 ± 305.48 CFU/100 mL. Air and water temperatures as well as conductivity only had two readings each, and pH, DO, and bacteria counts each had five total readings. Little Lotts Creek had the highest temperatures for both air and water averaging at 32.99 ± 3.46 °C and 25.45 ± 4.17 °C, respectively. The total pH level averaged at 6.9 ± 0.31, and Little Lotts dissolved oxygen was the highest among all sites at 7.7 ± 0.58 mg/L. The conductivity values for Little Lotts Creek were 128.75 ± 5.30 µS/cm. 

### 3.3. Trash Analysis

**Figure 13:**
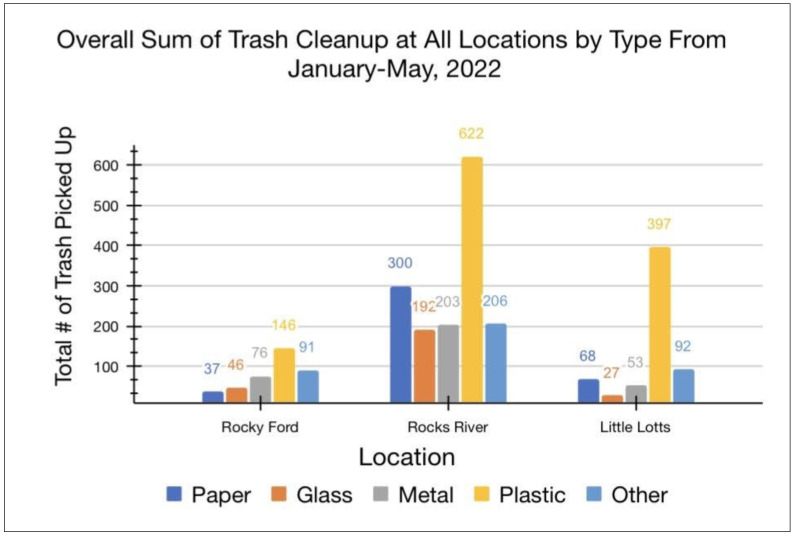
Trash Analysis—Total Overall Count of Trash Picked Up by Type per Location.

**Figure 14:**
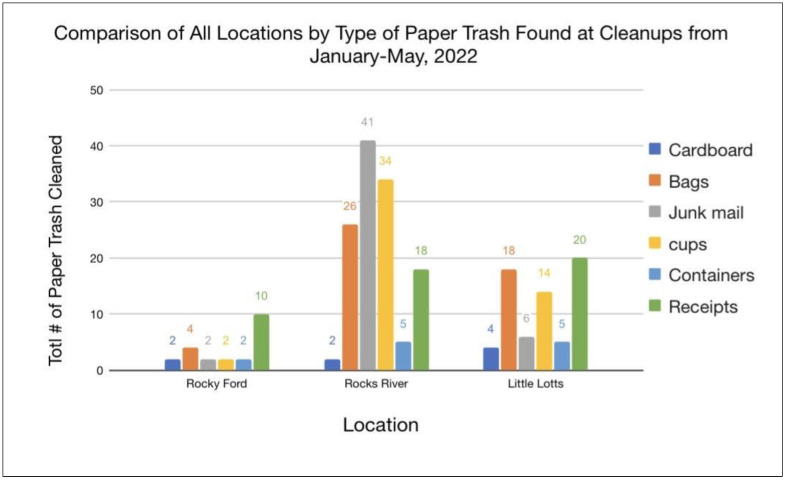
Trash Analysis—Paper Products Found During Cleanups.

**Figure 15:**
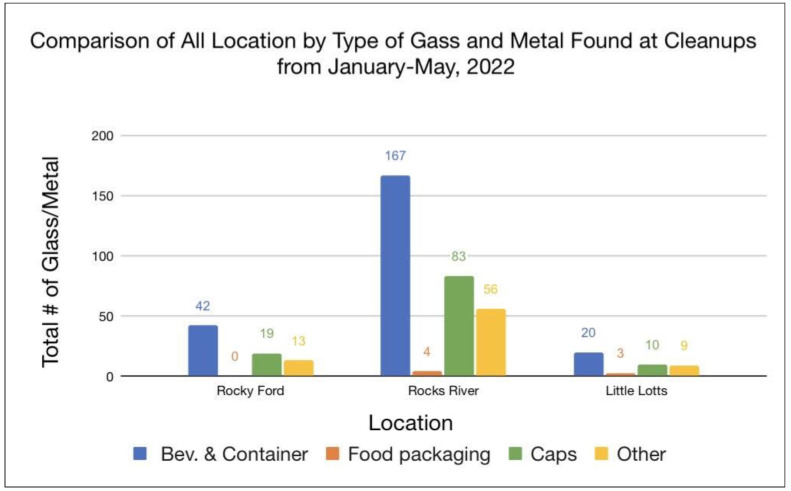
Trash Analysis—Glass and Metal Products Found During Cleanups.

**Figure 16:**
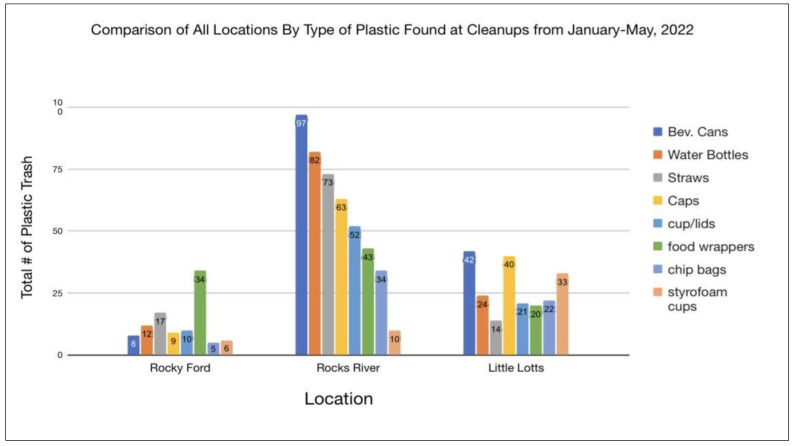
Trash Analysis—Plastic Products Found During Cleanups.

**Figure 17:**
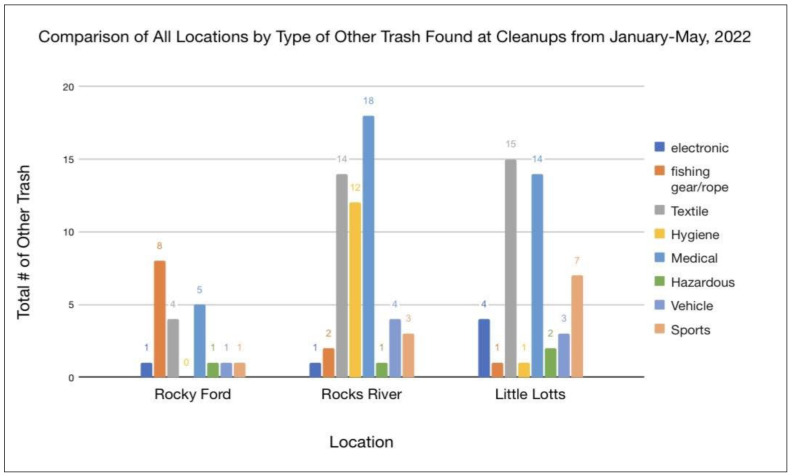
Trash Analysis—Other Commonly Found Trash during Cleanups.

In Figure 13, average monthly trash cleaned up by the pound is shown for each location. Rocks River had a total of 19.62 kg cleaned from its site with the least amount of trash collected. Little Lotts Creek had the second highest amount of trash picked up with 48.84 kg followed by Rocks River Landing with a total of 69.34 kg picked up throughout the study. After each cleanup, a trash analysis form was completed to help provide information as to what exactly was found at these locations. This form was beneficial in helping to separate the trash into categories based on type. These categories were paper, glass, metal, plastic, and other if it did not fit into the other categories and is represented in Figure 13 with each location also shown. Each of these categories are then broken down further in Figure 14, Figure 15, Figure 16 and Figure 17. Although Rocky Ford had the least amount of trash, the number of plastic trash found at this landing was 146 pieces. Plastic was the most commonly found type of trash at every location followed by the category considered “other” and the paper category. At Little Lotts Creek, 397 pieces of plastic trash was found; meanwhile, at Rocks River Landing, 622 pieces of plastic trash were found, along with 300 pieces of paper trash, 206 pieces of trash under the “other” category, 203 pieces of metal, and 192 pieces of glass. 

Under the paper category in Figure 14, six labels were created to represent it, including cardboard, bags, junk mail, cups, containers, and receipts. Receipts were most common at Rocky Ford Landings. Junk mail was followed by cups, bags, and receipts that were most commonly found at Rocks River with numbers 41, 34, 26, and 18 picked up and had the most paper trash overall. A total of 20 receipts, 18 bags, and 14 paper cups were collected at Little Lotts and had the second highest amount of paper trash compared to all sites. Glass and metal were combined in Figure 15 due to the low numbers. Rocks river once again had the highest number count overall with 167 pieces of beverage and containers, 83 bottled caps, 56 other types of glass and metal including shards and broken pieces of unknown products, and 4 food packaging products. Rocky Ford Landing had the second highest amount of glass and metal with 42 beverages and containers, 19 bottled caps, and 13 other types of glass and metal with no glass or metal food packages. Little Lotts Creek had the least, which could be because most trash was picked up from the surface of the water versus the on land when comparing the other site locations. For beverages and containers only 20 pieces were found, 10 bottled caps, 9 “other”, and 3 metal or glass food packages. 

Plastic trash was by far the highest amount of trash found from all locations and is illustrated in Figure 16. The plastic category was split into eight new categories being beverage containers, water bottles, straws, bottled caps, cups/lids, food wrappers, chip bags, and Styrofoam cups. At Rocky Ford, where the least amount of plastic was found, only 34 food wrappers, 17 straws, 12 water bottles, 9 bottled caps, 8 beverages containers, 6 Styrofoam cups, and 5 chip bags were found. Rocks River had the most plastic trash with its top five products being beverage containers, water bottles, straws, caps, and cups/lids with numbers of 97, 82, 73, 63, and 52 being collected, respectively. Finally, at Little Lotts Creek, its top three plastic products were 42 beverage containers, 40 bottled caps, and 33 Styrofoam cups and pieces being collected. The category named “other” also had eight categories to help dissect the trash found at these locations and is represented in Figure 17. The products in this category are electronics, fishing gear/rope, textile, hygiene products, medical, hazardous, vehicle related, and sports equipment. One category not represented in this Figure is tobacco, since the number of cigarettes and containers that were found did not fit into the Figure. At Rocks River Landing, 356 cigarette butts were picked up followed by Little Lotts with 148 cigarette butts, and Rocky Ford at 70 cigarette butts cleaned up from these sites within 5 months. Rocks River Landing and Little Lotts had the most products found in this category. Rocks River’s top three products were electronics such as cell phones, earbuds, and circuits with 18, textiles such as clothes, shoes, and household fabrics with 14, and 12 hygiene products. Little Lotts Creek’s top three included 15 textiles, 14 electronics, and 7 sport equipment products. Rocky Ford had the lowest number of products in this category. The top three items found at Rocky Ford Landing were 8 fishing gear/rope, 5 electronics, and 4 textiles.

## 4. Discussion

Air contamination was most impacted at Little Lotts Creek and Rocks River Landing, where trash was also collected the most. From gasses to bacterial colony-forming unit counts, these two locations were dramatically higher than Rocky Ford Landing. Most of Rocks River trash was found along the riverbed and up further on the Landing itself, allowing plenty of open area to collect trash. With no trash cans in sight for visitors to throw away their food waste and with little cleanup, plastic and paper became overwhelmingly high at this location. Plastic was highest at all locations; however, Rocks River Landing stood out among the other locations. Plastic does not decompose into the soil or degrade at a fast rate. Past studies have found a positive correlation between high temperatures and low humidity to the release of gasses from plastic into the atmosphere, specifically methane and VOCs [14]. Another study by Jain et al. [15] also indicated that higher temperatures melted plastic at faster rates, further increasing the amount of gasses released. In fact, many of the plastics found at all sites were prone to have a chemical known as Bisphenol, which in low doses can leach into foods and water over time and are carcinogenic, cause insulin resistance and interfere with the reproductive system [15]. Trends seen from the two sites during early to late afternoon, where temperatures averaged anywhere from 21–27 °C, do show a positive correlation with the results from this study. Rocky Ford samples were taken in the morning where shade was usually provided. 

Water quality trends and floatable trash are noted throughout many research articles, noting the damages and impact that trash can have on wildlife, visibility, and visuality. Between Rocky Ford and Rocks River, the landing most impacted throughout this study was Rocks River along the Canoochee River. This was not likely due to trash as there was little trash located in the water specifically; however, beer, alcohol, and tobacco products lined the riverbed each week. The popularity of this particular landing also brought many fishermen and boaters. It was common to see an oily sheen floating along the surface of the water. There are not many notable studies to illustrate the impact that trash has on water quality in terms of conductivity or pH; however, some studies suggest that trash may have a microbiological impact. In terms of parameters, dissolved oxygen would be most impacted by trash. Davies-Colley et al. explains that “oxygen is usually of greatest concern because of its oxidizing role and crucial support of the respiration of aquatic life” [16]. Rocks River and Rocky Ford had similar trends throughout the study, but Rocks River Landing showed lower counts of dissolved oxygen than Rocky Ford Landing. 

Another study in California suggested that trash pollution has become an increasing problem with farmers and irrigation systems due to a lack of infrastructure and waste management [17]. The most common trash impacting farmers in California were metal, glass objects, trash bags full of trash, and animal and human waste such as sanitary napkins and diapers; however, 58% of all trash found by farmers was plastic [17]. Plastic was the most common among all the trash found throughout our research and was very prominent in other studies as well. In a study by Fork et al., microplastics were found as a major nuisance in the state of Pennsylvania as an emerging contaminant. Microplastics are generally introduced through trash in stormwater systems, wastewater treatment plants, or through fallen debris and can pose harm to animals and humans alike by leaching toxins into the environment and containing surfaces for harmful microbes to grow on and attach to [18]. 

Israt et al. illustrates that methane gas is a known cause of accumulated trash when anaerobic breakdown of litter takes place under the right conditions, specifically during the decaying process of organic materials [19]. The trash found in this study was mostly plastic and household waste, and the same results were shown in the study of Talley et al. [20], where bags and packaging waste had the highest percentage found in four different cities of California. The study also showed a significant increase in trash after rainy seasons with bags and packaging leading in the overall total [20]. The findings of this study are important to environmental and public health as trash enters the oceans through our rivers and creeks. This is not a local issue in South Georgia. Trash pollution impacts many rivers and creeks throughout the United States. As pollution continues to accumulate and fill our oceans, other countries are also impacted. In fact, about 80%, or two-thirds, of all ocean garbage comes from an average of 1000 rivers [21]. This is a huge issue for wildlife, drinking water, and agricultural irrigation systems.

There were several limitations to this study, including that the popularity of each site could have affected the amount of trash found. Little Lotts Creek was in an urban location and had volunteers once a month when trash was collected, improving the chances of collecting a greater amount of trash, versus Rocky Ford Landing and Rocks River Landing whose trash was collected by one person for an average of one hour each time. Chemical and bacterial data for water quality were not taken on the same days as air quality data since other Ogeechee Riverkeeper interns and volunteers were already assigned to take samples at this location. This also affected not having the same number of samples which could have affected the outcome. There were no water data available for the month of January for all sites, and none for the river landings for February due to missing chemicals from the kit, out-of-date chemicals, and not having an Adopt-A-Stream water monitoring certificate until 5 February 2022. For the first week of sampling, no ammonia instrument was available to take samples, but was available for the remainder of the study. This study was approximately five months long; however, the data collected for all parameters were not consistent and do not offer a long enough time frame to see how trash may impact both air and water quality. 

More research on plastics, glass, metal cans, and other litter will need to be conducted in order to determine the effects on air and water quality pollutions. Bi-weekly and monthly averages of trash cleaned up from all sites noted that Little Lotts Creek had the highest averages most months, followed by Rocks River and then Rocky Ford Landing. Future studies could possibly analyze trash impact in water and air on a smaller scale by producing lab studies through close daily monitoring and placing trash under different conditions. Researchers may also decide to have one location as a focal point versus comparing several locations to reduce human error, outside factors, and other unforeseen confounders. 

## 5. Conclusions

The potential contamination of river landings and creeks may contribute to increased levels of airborne and waterborne gas levels and microbial loads near the river water surfaces. Creeks compared to rivers seemed to be more concentrated and had higher concentrations of air pollution and water pollution and may be more severe with concerns to public health. Since most trash for the river landings was found on land versus in the water, it was not surprising that the air quality was worse at Rocks River Landing than at Little Lotts Creek, whereas the water quality was worse in Little Lotts Creek than at the river landings.

## Figures and Tables

**Table 1:** Total Overall Air Quality Averages from All Sites. Total Air Quality Averages and Standard Deviations from January–May 2022.

Location	Temperature (°C)	Relative Humidity (%)	Ammonia (ppm)	Methane (ppm)	VOCs (ppm)	Bacteria (CFU/m^3^)
Rocky Ford	17.12 ± 2.75	61.0 ± 16.6	2.3 ± 2.3	2.7 ± 3.3	0.19 ± 0.13	407.7 ± 427.3
Rocks River	21.83 ± 6.42	48.0 ± 16.4	3.7 ± 3.3	5.1 ± 4.7	0.31 ± 0.18	634.9 ± 851.9
Little Lotts	27.33 ± 6.32	38.5 ± 11.6	5.3 ± 3.8	5.6 ± 3.5	0.38 ± 0.24	666.3 ± 1034.1

**Table 2:** Water Quality—Water Sampling Parameter Dates and Measurements for Little Lotts Creek. Average Water Sampling Measurements from Little Lotts Creek.

Date	Air Temperature (°C)	Water Temperature (°C)	pH	Dissolved Oxygen (mg/L)	Conductivity (µS/cm)	*Escherichia coli* (CFU/100 mL)
19 February 2022	-	-	7.25	8.4	-	-
21 February 2022	-	-	-	-	-	99.5
6 March 2022	-	-	6.5	7	-	500.00
8 March 2022	-	-	-	-	-	766.00
11 April 2022	35.44	22.5	7	7.6	125	150.00
17 May 2022	30.55	28.4	7	7.8	132.5	66.00

**Table 3:** Total Water Parameter Averages and Standard Deviations for All Sites. Total Water Parameter Averages and Standard Deviations from January–May 2022.

Location	Air Temperature (°C)	Water Temperature (°C)	pH	Dissolved Oxygen (mg/L)	Conductivity (µS/cm)	*Escherichia coli* (CFU/100 mL)
Rocky Ford	20.69 ± 3.8	18.16 ± 3.1	7 ± 0.00	6.16 ± 0.4	143.0 ± 33.6	193.2 ± 79.4
Rocks River	28.09 ± 3.58	21.27 ± 3.48	6.5 ± 0.40	6.4 ± 0.82	80 ± 44.54	233.33 ± 50.85
Little Lotts	32.99 ± 3.46	25.45 ± 4.17	6.9 ± 0.31	7.7 ± 0.58	128.75 ± 5.30	316.3 ± 305.48

## Data Availability

Data collected in this study are all contained within the article.
